# Electroacupuncture Ameliorates Premature Ovarian Insufficiency by Inhibiting METTL3‐Mediated m6A Methylation of Beclin 1 mRNA in Granulosa Cells

**DOI:** 10.1002/fsn3.71697

**Published:** 2026-06-21

**Authors:** Lele Ling, Sining He, Xue Zhao, Yaran Sheng, Mengying Hong, Long Yuan, Peng Liu, Boliang Ke, Bingrong Li, Bimeng Zhang

**Affiliations:** ^1^ Department of Obstetrics and Gynecology Shanghai Sixth People's Hospital Affiliated to Shanghai Jiao Tong University School of Medicine Shanghai China; ^2^ Shanghai University of Traditional Chinese Medicine Shanghai China; ^3^ Department of Acupuncture, Shanghai General Hospital Shanghai Jiao Tong University School of Medicine Shanghai China; ^4^ Department of Geriatrics Affiliated Longhua Hospital of Shanghai University of Traditional Chinese Medicine Shanghai China; ^5^ School of Traditional Chinese Medicine Shanghai University of Traditional Chinese Medicine Shanghai China; ^6^ Department of Traditional Chinese Medicine Sheshan Town Community Health Service Center of Songjiang District Shanghai China; ^7^ Department of General Practice Songjiang Hospital Affiliated Shanghai Jiao Tong University School of Medicine Shanghai China

**Keywords:** autophagy, electroacupuncture, granulosa cells, high‐fat high‐sugar (HFHS) diet, N6‐methyladenosine methylation, premature ovarian insufficiency

## Abstract

Premature ovarian insufficiency (POI) is a major cause of infertility in young women. In this study, we investigated the therapeutic effects of electroacupuncture (EA) on ovarian function and granulosa cell autophagy in a high‐fat, high‐sugar (HFHS) diet–induced POI mouse model. POI was established by HFHS feeding, and mice received EA stimulation at CV4, ST36, and SP6. Ovarian morphology was evaluated using hematoxylin and eosin (HE), Masson's trichrome, and Sirius Red staining. Estrous cycles were monitored by crystal violet staining, and serum sex hormones and inflammatory cytokines were measured by ELISA. Cholesterol accumulation, apoptosis, and FSHR expression were assessed using filipin staining, TUNEL assays, and immunofluorescence, respectively. Single‐cell RNA sequencing was performed to characterize ovarian cell subpopulations and autophagy‐related transcriptional signatures. Autophagosome formation and the expression of autophagy‐related markers (Beclin1, LC3, and P62), as well as METTL3, were analyzed by transmission electron microscopy (TEM), RT‐qPCR, Western blotting, and immunohistochemistry. m6A modification levels on Beclin1 mRNA were determined using LC–MS, colorimetric assays, and RIP‐qPCR. The results demonstrated that EA significantly improved follicular development, normalized estrous cycles, reduced granulosa cell apoptosis, and decreased inflammatory cytokine levels in POI mice. Single‐cell analysis revealed that granulosa cells exhibited the highest autophagy activity and were strongly associated with senescence‐related pathways. EA markedly reduced autophagosome formation, downregulated Beclin1 and LC3 expression, and upregulated P62, indicating suppression of excessive autophagy. Furthermore, EA decreased METTL3 expression and reduced m6A modification of Beclin1 mRNA, thereby limiting Beclin1 translation. Collectively, these findings suggest that EA ameliorates HFHS‐induced POI by inhibiting METTL3‐mediated m6A methylation of Beclin1 mRNA, suppressing excessive granulosa cell autophagy, and restoring ovarian function, thereby revealing a potential epigenetic mechanism underlying the therapeutic effects of EA in diet‐related ovarian dysfunction.

## Graphical abstract

A high‐fat, high‐sugar (HFHS) diet induces premature ovarian insufficiency (POI), characterized by reduced follicular reserve, increased atresia, fibrosis, and excessive granulosa cell autophagy. Electroacupuncture (EA) at CV4, ST36, and SP6 suppresses METTL3 expression and reduces m6A methylation of Beclin1 mRNA, thereby limiting Beclin1 translation and alleviating excessive autophagy. Through regulation of the METTL3‐mediated m6A modification of Beclin1, EA restores autophagy balance, improves granulosa cell survival, and promotes recovery of ovarian function. The figure was created with BioRender.com.

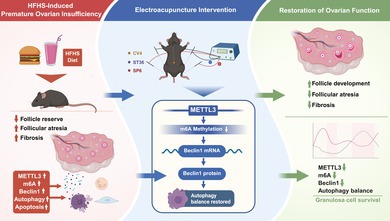

## 1 Introduction

In recent years, dietary patterns have emerged as significant environmental factors affecting human reproductive health. Modern high‐fat, high‐sugar (HFHS) diets—characterized by excessive intake of saturated fats, refined carbohydrates, fructose syrups, and red meat, accompanied by inadequate consumption of vegetables and fruits—are increasingly prevalent. This nutritional imbalance not only predisposes individuals to obesity but also serves as a key trigger for metabolic syndromes (e.g., hyperlipidemia, insulin resistance, type 2 diabetes) and infertility (Rejani et al. 2022; Varlamov 2017). Research indicates that HFHS diets impair female reproductive function through multiple mechanisms: saturated fatty acids induce lipotoxicity, disrupting cellular integrity (Bergman and Ader 2000; Wajchenberg 2000), while high sugar intake exacerbates insulin resistance, hepatic lipid deposition, and systemic inflammation, further compromising reproductive capacity (Hatch et al. 2018; Stanhope 2016). The ovary, as a highly metabolically active organ, is particularly vulnerable to these metabolic disturbances, which manifest as enhanced oxidative stress, mitochondrial dysfunction, and abnormal follicular development (Smits et al. 2021; Marei et al. 2020). These alterations ultimately accelerate follicular reserve depletion and hasten ovarian functional decline (Liu, Lin, et al. 2021; Liu, Jing, et al. 2021).

Premature ovarian insufficiency (POI) is a leading cause of infertility in women under 40, with a prevalence of approximately 2.8% in the female population in China and a trend toward earlier onset (Wu et al. 2014). Beyond infertility, POI carries systemic complications such as osteoporosis, cardiovascular diseases, mood disorders, and reduced life expectancy (Chon et al. 2021; Shareghi‐Oskoue et al. 2021). Current clinical management relies primarily on hormone replacement therapy (HRT) to alleviate hypoestrogenic symptoms (Machura et al. 2018; Madalinska et al. 2006), though long‐term HRT use may increase risks of breast cancer, stroke, and thrombosis (Nelson et al. 2002; Barrett‐Connor and Stuenkel 2001). In assisted reproduction, oocyte donation improves conception rates but is subject to ethical controversies and potential genetic risks (Klein and Sauer 2010; Klitzman and Sauer 2015). Emerging techniques—including stem cell transplantation, platelet‐rich plasma (PRP), and in vitro activation (IVA) of follicles—show preliminary promise for POI treatment, yet their safety profiles and applicability require further validation (Wang et al. 2021; Ding et al. 2023). Consequently, developing safe, effective, and mechanistically clarified interventions remains pivotal in POI research.

Electroacupuncture (EA), a therapy integrating traditional acupuncture with modern electrical stimulation, offers advantages such as controllable intensity, operational simplicity, and minimal adverse effects. It is widely applied in metabolic disorders and gynecological conditions (Fu et al. 2020). Clinical studies demonstrate that EA reduces waist circumference and body fat in central obesity, with some female patients exhibiting menstrual cycle improvements, suggesting its potential for endocrine regulation (Lam et al. 2024). Previous research indicates that EA modulates hypothalamic–pituitary‐ovarian (HPO) axis function and improves serum sex hormone levels via neuroendocrine mechanisms (Zhu et al. 2019). Our group has further confirmed that EA stimulation at the Guanyuan (CV4), Zusanli (ST36), and Sanyinjiao (SP6) acupoints elevates antioxidant levels, such as glutathione (GSH) and superoxide dismutase (SOD), alleviates HFHS‐induced ovarian oxidative stress, and preserves ovarian function (Geng et al. 2022). Additionally, combined EA and HRT significantly enhances pregnancy rates while mitigating HRT‐related side effects (Yang et al. 2023), highlighting the therapeutic synergy of acupuncture and pharmacotherapy for POI.

Follicular development critically depends on granulosa cells, which orchestrate steroidogenesis, oocyte nourishment, and local endocrine signaling. Granulosa cell dysfunction is recognized as a central mechanism in POI pathogenesis (Robker et al. 2018; Hsueh et al. 2015). Autophagy, a key homeostatic process, supports follicular development by eliminating damaged cellular components when moderately activated; however, excessive autophagy may accelerate follicular atresia and follicular pool exhaustion, leading to ovarian functional decline (Dai et al. 2023; Wu et al. 2022). Investigations into EA‐regulated autophagy mechanisms reveal that acupuncture modulates this process across disease models—e.g., by activating the mTOR pathway in Parkinson's disease (Ning et al. 2023) or regulating TSC1‐mTOR demethylation in metabolic disorders (Zhang, Lee, et al. 2021). While the role of granulosa cell autophagy in POI has been documented, its precise regulatory mechanisms, particularly regarding the molecular basis of EA‐mediated autophagy control, remain poorly understood.

Recent advances in epigenetics, especially RNA modification, offer novel insights into granulosa cell regulation. Among these, N6‐methyladenosine (m6A)—the most abundant mRNA modification—is dynamically regulated by “writer” methyltransferases, “eraser” demethylases, and “reader” recognition proteins (Fu et al. 2014). m6A participates in ovarian development, oocyte maturation, and granulosa cell function, particularly in autophagy, stress responses, and cell fate determination (Qiao et al. 2014; Wang et al. 2020). Studies report that m6A modifications, mediated by enzymes such as METTL3 and FTO, significantly influence granulosa cell autophagy (Li et al. 2024; Li et al. 2023). Our recent work shows that EA downregulates ac4C acetylation of P16 mRNA and NAT10 expression in POI mouse ovaries (Geng, Liu, et al. 2023), suggesting EA improves the ovarian microenvironment via epigenetic mechanisms. However, the dynamics of m6A modifications in HFHS‐induced POI and their role as therapeutic targets for EA remain unexplored.

This study employs an HFHS‐induced POI mouse model to evaluate EA's effects on ovarian structure, hormone levels, inflammatory status, and estrous cyclicity. Utilizing Single‐cell RNA sequencing (scRNA‐seq), Western blotting, immunohistochemistry, Liquid chromatography‐mass spectrometry (LC–MS), and MeRIP‐qPCR, we investigate how EA modulates METTL3‐mediated m6A methylation of Beclin1 mRNA and its mechanistic role in granulosa cell autophagy. By elucidating EA's molecular basis for treating POI, this work aims to advance theoretical support and novel therapeutic targets for traditional Chinese medicine in ovarian aging, facilitating clinical translation of acupuncture‐based interventions.

## 2 Materials and Methods

### 2.1 Experimental Animals

Female C57BL/6 mice (SPF grade), aged 6–8 weeks and weighing approximately 20 ± 2 g, were sourced from the Animal Experiment Center of the First People's Hospital affiliated with Shanghai Jiao Tong University School of Medicine, animal ethics approval number: 2020AW126. The mice were maintained in barrier facilities utilizing individually ventilated cage systems under controlled conditions: temperature (22°C ± 1°C), humidity (55% ± 5%), and a 12‐h light/dark cycle. Sterilized feed and acidified water (pH 2.5–3.0) were provided ad libitum. All procedures adhered to animal ethics standards and received approval from the institutional ethics committee.

### 2.2 POI Animal Model

Following a 7‐day acclimatization period, the mice were randomly allocated into three groups (*n* = 15 per group): wild‐type (WT), POI model, and EA intervention. The POI and EA groups received a daily high‐fat diet (8 g/kg) and intragastric administration of a 30% lactulose solution (200 μL) for 8 weeks, while the WT controls were fed a standard diet and received an equal volume of saline (Liu, Lin, et al. 2021; Liu, Jing, et al. 2021; Zhu et al. 2021). Successful modeling of POI was defined by the following criteria: (1) elevated serum FSH and decreased E2 levels; (2) histopathological evidence of reduced follicular development and increased atretic follicles (Mo et al. 2024; Geng, Nie, et al. 2023).

### 2.3 EA Treatment

In accordance with standardized mouse acupoint guidelines (China Association of acupuncture and Moxibustion 2025), the restrained mice in the EA group received needle insertion at the Guanyuan (CV4), bilateral Sanyinjiao (SP6), and Zusanli (ST36) acupoints. Sterile needles (0.18 mm × 13 mm) were inserted vertically to a depth of 1–2 mm and manually stimulated through lifting, thrusting, and rotation for 1 min. Subsequently, the needles were connected to an SDZ‐V electroacupuncture device, delivering a continuous wave at 2 Hz and an intensity of 0.1–1 mA for 30 min. The treatment intensity was calibrated to elicit localized tremors without causing vocalization or agitation. EA treatment was administered every other day for 4 weeks. Mice in the WT and POI groups underwent identical restraint procedures without electroacupuncture.

### 2.4 Histological Analysis and Follicle Counting

Fixed ovaries were dehydrated, paraffin‐embedded, sectioned, and dewaxed in xylene. Rehydration proceeded through graded ethanol to distilled water. Sections were stained with hematoxylin (Beyotime, C0105M, China) for 5 min, rinsed, differentiated in 1% acid alcohol, counterstained with eosin for 15 s, rinsed again, dehydrated through graded ethanol, and cleared in xylene. Mounted sections (Leica RM2235, Germany) were imaged under a light microscope (Nikon Eclipse Ci‐L, Japan). Follicle counting was performed in every fifth serial section to avoid double counting of the same follicle. Follicles were classified according to established morphological criteria as primordial, primary, secondary, and antral follicles. Only follicles containing a clearly visible oocyte nucleus were included in the analysis. Atretic follicles were identified based on the presence of granulosa cell disorganization, pyknotic nuclei, or oocyte degeneration and were recorded separately. Follicle numbers were quantified in a blinded manner by two independent investigators. The average number of follicles per ovary was calculated for statistical analysis.

### 2.5 Estrous Cycle Monitoring

Daily vaginal smears were collected between 08:00 and 10:00 for ten consecutive days. Using sterile saline‐moistened swabs, secretions were gently obtained from the vaginal canal and transferred to glass slides. After air‐drying, smears were stained with crystal violet for 1–2 min, rinsed with distilled water, and dried. Estrous stages were determined by examining cellular morphology under light microscopy.

### 2.6 Masson Staining

Dewaxed and rehydrated sections were triple‐stained according to kit protocols (Beyotime, Shanghai, China; Cat# C0189M). Briefly: sections were stained with hematoxylin (50 μL), differentiated in acid solution (30 s), and rinsed with distilled water; treated with Ponceau‐acid fuchsin (50 μL, 10 min), differentiated in phosphomolybdic acid (2 min), and rinsed; stained with bright green solution (50 μL, 1 min), and differentiated in acid solution (1 min). Sections were subsequently dehydrated, cleared in xylene, mounted, and imaged.

### 2.7 Sirius Red Staining

Dewaxed sections were stained with Sirius Red/picric acid solution (Abcam, Cambridge, UK; Cat# ab150681) for 60 min under dark conditions, rinsed three times in absolute ethanol (10 s per rinse), cleared in xylene, and mounted. Collagen fibers were identified by bright red birefringence under light microscopy.

### 2.8 Enzyme‐Linked Immunosorbent Assay (ELISA)

Serum follicle‐stimulating hormone (FSH), estradiol (E2), interleukin‐1β (IL‐1β), and tumor necrosis factor‐α (TNF‐α) levels were quantified using commercial ELISA kits (Geneme Biotechnology, Shanghai, China; Cat# JM‐02838M1, JM‐02849M2, JM‐02323M1, and JM‐02415M1, respectively). Microplates included standard, sample, and blank wells. Following reaction termination, absorbance at 450 nm was measured within 15 min using a microplate reader (BIO‐RAD 550, Hercules, CA, USA), with blank wells serving as the zero reference.

### 2.9 Filipin Staining

Dewaxed and rehydrated sections were processed according to kit instructions (Genomeditech, Shanghai, China; Cat# GMMS80079.2.1). Briefly: 500 μL dewaxing solution covered sections, incubated at 70°C for 5 min, and residual solution was drained. Sections were sequentially treated with 200 μL purification solution (2 min, room temperature [RT]), 200 μL clearing solution (3 min, RT), and 200 μL blocking solution (10 min, RT), with each solution carefully drained after incubation. Under dark conditions, 100 μL staining solution was applied for 30 min (RT) to bind free cholesterol. Sections were rinsed with 200 μL clearing solution, mounted, and analyzed via fluorescence microscopy for cholesterol‐positive (bright blue) signals.

### 2.10 TUNEL Staining

Dewaxed and rehydrated sections were treated with 20 g/mL DNase‐free proteinase K at 37°C for 15 min, rinsed with PBS, and incubated with 50 μL TUNEL detection reagent (Beyotime, Cat# C1086) in the dark (37°C, 60 min). After three 5‐min washes in PBS, sections were mounted with anti‐fade medium and imaged via fluorescence microscopy to assess granulosa cell apoptosis.

### 2.11 Immunofluorescence Staining

Dewaxed sections underwent antigen retrieval twice, were blocked with 5% bovine serum albumin (BSA) at 37°C for 1 h, and incubated with primary antibodies overnight at 4°C. Primary antibodies included: anti‐FSHR (Proteintech, Rosemont, IL, USA; Cat# 22665‐1‐AP; 1:200). Following three 5‐min washes with Tris‐buffered saline with Tween‐20 (TBST), sections were incubated with secondary antibodies (goat anti‐rabbit IgG Alexa Fluor555, ab150078) at 37°C for 1 h. Sections were mounted with 4′,6‐diamidino‐2‐phenylindole (DAPI)‐containing medium (Beyotime, Shanghai, China; Cat# P0131) and imaged.

### 2.12 10x Single‐Cell Processing and cDNA Library Preparation

Ovarian tissues were preserved in MACS Tissue Storage Solution (Miltenyi Biotec, Bergisch Gladbach, Germany; Cat# 130‐100‐008) immediately post‐dissection. Single‐cell suspensions were encapsulated with gel beads in oil droplets (GEMs) using the Chromium X platform (10× Genomics, Pleasanton, CA, USA). Messenger RNA (mRNA) released from lysed cells bound barcoded primers for complementary DNA (cDNA) synthesis. Purified cDNA was amplified to construct sequencing libraries.

### 2.13 scRNA‐Seq Analysis

Data were processed using Seurat (version 4.3.0). Quality control thresholds were applied as follows: nFeature_RNA < 5000, mitochondrial gene percentage < 5%, ribosomal gene percentage > 3%, and hemoglobin gene percentage < 0.1%. After filtering (retaining 24,478 genes across 41,513 cells), data were normalized using the ScaleData function, dimensionality‐reduced via principal component analysis (PCA), and batch‐effect corrected using Harmony. Clustering (resolution = 0.3) and uniform manifold approximation and projection (UMAP) were performed for visualization. Cell types were manually annotated based on established marker genes.

### 2.14 Transmission Electron Microscopy (TEM)

Ovaries from three mice per group were fixed in 2.5% glutaraldehyde (Servicebio, Wuhan, China; Cat# G1102) at room temperature for 30 min, then stored overnight at 4°C. Tissues were rinsed in phosphate‐buffered saline (PBS), post‐fixed in 1% osmium tetroxide, dehydrated in ethanol, embedded in resin, and sectioned (40–50 nm thickness). Uranyl acetate‐stained sections were imaged using an FEI Tecnai G2 F20 transmission electron microscope (Thermo Fisher Scientific, Hillsboro, OR, USA).

### 2.15 Immunohistochemistry (IHC)

Antigen‐retrieved sections were blocked with 3% hydrogen peroxide at room temperature for 10 min, followed by goat serum (Beyotime, Cat# C0265) for 10 min. Primary antibodies—anti‐LC3A/B (Affinity, Cat# AF5402), anti‐Beclin1 (Cell Signaling Technology, Danvers, MA, USA; Cat# 3738), and anti‐P62 (Cell Signaling Technology; Cat# 5114)—were applied overnight at 4°C. After PBS washes, sections were incubated with horseradish peroxidase (HRP)‐conjugated secondary antibody (Abcam, Cat# ab205718) at room temperature for 10 min, treated with streptavidin‐peroxidase for 10 min, and developed with 3,3′‐diaminobenzidine (DAB; incubation: 30 s–3 min). Sections were counterstained with hematoxylin, dehydrated, mounted, and analyzed via light microscopy.

### 2.16 Western Blot

Following protein extraction, ovarian tissue was washed with ice‐cold PBS and cut into 1 mm^3^ pieces. Approximately 20 mg of tissue was added to 60 μL of RIPA lysis buffer, homogenized to achieve lysis, and then centrifuged at 10000–14000 g for 3–5 min at 4°C to collect the supernatant. Protein quantification was subsequently performed using the BCA method (Epizyme Biotech Co. Ltd., Cat# ZJ102L, Shanghai, China), where the A562 value was measured using a 96‐well plate and the concentration was calculated based on a standard curve. Protein samples, after being mixed with loading buffer and boiled at 100°C for 10 min, were loaded onto self‐prepared SDS‐PAGE gels. A dual‐color pre‐stained protein marker was used to estimate molecular weights. Electrophoresis was first conducted at 80 V for 30 min, followed by protein separation at 120 V for 1.5 h. After electrophoresis, proteins were transferred onto PVDF membranes activated with methanol, using a transfer cassette assembled with WB‐specific transfer sponges, under 200 mA current for 2 h. The membranes were then blocked with a no‐protein fast blocking buffer for 10 min. Subsequently, the membranes were incubated with primary antibodies (Anti‐LC3A/B antibody, Affinity Biosciences, Cat# AF5402, USA; Anti‐Beclin 1 antibody, CST, Cat# 3738, USA; Anti‐P62 antibody, CST, Cat# 5114, USA; Recombinant Anti‐METTL3 antibody, Abcam, Cat# ab195352, UK; Anti‐Beclin 1 antibody, Affinity Biosciences, Cat# AF5128, USA) placed in a humidified chamber overnight at 4°C. This was followed by incubation with the secondary antibody (Goat Anti‐Rabbit IgG H&L (HRP), Abcam, Cat# ab205718, UK) at room temperature for 1 h. Enhanced ECL chemiluminescent substrate kit (Vazyme Biotech Co. Ltd., Cat# E411‐04, Nanjing, China) was used for detection, and band images were captured and analyzed using either a Tanon 4600 imaging system (Tanon Life Science Co. Ltd., Model Tanon 4600 SF, Shanghai, China) or a gel imager (BIO‐RAD, Model 2500, USA).

### 2.17 RT‐qPCR

RNA extraction was first performed by grinding 10–20 mg of ovarian tissue (approximately the size of a grain of rice) in 500 μL of Lysis Buffer until complete lysis was achieved, followed by vigorous vortexing for 30 s and incubation at room temperature for 5 min, after which the lysate was centrifuged at 12,000 rpm for 5 min to collect the supernatant. The supernatant was then passed through HyperPure gDNA‐Filter Columns placed in collection tubes and centrifuged at 12,000 rpm for 5 min to remove genomic DNA and impurities. An equal volume of absolute ethanol (approximately 250 μL) was added to the supernatant, mixed thoroughly, and transferred to HyperPure RNA Columns in collection tubes. Sequential washes were performed using 500 μL of Wash Buffer A followed by two washes with 500 μL of Wash Buffer B, with each wash step involving centrifugation at 12,000 rpm for 30 s at room temperature. After a final 2‐min centrifugation of the empty column to remove residual ethanol, RNA was eluted using 50–100 μL of Elution Water. For the reverse transcription step, cDNA synthesis was carried out according to the HyperScript III RT SuperMix for qPCR kit protocol (EnzyArtisan Biological Co. Ltd., Cat# R102‐02, Shanghai, China). A 20 μL reaction mixture was prepared containing 1 ng–5 μg of total RNA, 4 μL of 5× SuperMix, and RNase‐free water. The reaction proceeded at 37°C for 15 min, followed by enzyme inactivation at 85°C for 5 s. For quantitative PCR, reactions were assembled according to the 2× S6 Universal SYBR qPCR Mix protocol (EnzyArtisan Biological Co. Ltd., Cat# Q204‐01, Shanghai, China). A 20 μL reaction mixture was prepared containing 10 μL of 2× SYBR mix, 2 μL each of 10 μM forward and reverse primers, 2 μL of cDNA template, and 4 μL of ddH₂O. Amplification was performed on a QuantStudio 6 Flex Real‐Time PCR System (Thermo Fisher Scientific, Instrument Model, USA) using the following program: initial denaturation at 95°C for 30 s (1 cycle), followed by 40–45 amplification cycles of 95°C for 3–10 s and 60°C for 10–30 s, with a final melt curve analysis step using the instrument's default settings. Relative mRNA expression levels of target genes were calculated using the 2^(−ΔΔCT) method with 18S rRNA as the internal reference gene; primer sequences for target genes are listed in Table 1.

**TABLE 1:** Primer sequences of target genes.

Gene	Forward primer (5′→3′)	Reverse primer (5′→3′)
Mouse‐LC3B	GTCCTGGACAAGACCAAGTTCC	GAGGAAGAAGGCTTGGTTAGCA
Mouse‐Beclin1	GGAGGGGTCTAAGGCGTCCAG	TCTTGAAGCTCGTGTCCAGTTTCAG
Mouse‐METTL3	CACAACAGCCAAGGAACAGTCC	AATCACAAAATTCTTGCACCTGG
Mouse‐P62/SQSTM1	GCTCTTCGGAAGTCAGCAAACC	GCAGTTTCCCGACTCCATCTGT
Mouse‐18S rRNA	GTAACCCGTTGAACCCCATT	CCATCCAATCGGTAGTAGCG

### 2.18 m6A RNA Total Methylation Detection

Total m6A RNA methylation levels in mouse ovarian tissues were quantified using an m6A RNA methylation quantification kit (Abcam, Cambridge, UK; Cat# ab233491). Reagents were pre‐diluted as follows: 10× wash buffer (1:9 dilution to 1× working solution), capture antibody (1:1000), detection antibody (1:2000), and enhancer solution (1:5000). Positive control (0.5 ng/μL) and standards (0.01–0.50 ng/μL) were prepared in Tris‐EDTA (TE) buffer. For RNA binding, each well received 80 μL binding solution, 2 μL negative/positive controls, and sample RNA (100–300 ng total RNA), incubated at 37°C for 90 min, followed by three 2‐min washes. For m6A RNA capture, 50 μL capture antibody was added and incubated at room temperature for 60 min, washed; 50 μL detection antibody incubated at room temperature for 30 min, washed; and 50 μL enhancer incubated at room temperature for 30 min, washed. For colorimetric detection, 100 μL developer was added per well and incubated under dark conditions at room temperature until positive controls displayed medium blue coloration (5–10 min). Absorbance at 450 nm was measured after reaction termination.

### 2.19 LC–MS for mRNA Modifications

mRNA was isolated using NEBNext Poly(A) magnetic beads (New England Biolabs, Ipswich, MA, USA), followed by enzymatic digestion and dephosphorylation. After hydrolysis at 37°C for 3 h and purification, samples were analyzed using an Agilent 6460 triple quadrupole mass spectrometer coupled to a 1260 high‐performance liquid chromatography system (Agilent Technologies, Santa Clara, CA, USA).

### 2.20 MeRIP‐PCR

Total ovarian RNA was fragmented at 94°C for 5 min into 100–200 nucleotide segments. Fragmented RNA was incubated with m6A‐specific antibody (ABclonal, Woburn, MA, USA; Cat# A19841) at 4°C for 2 h with rotation. RNA‐antibody complexes were captured using Protein A/G magnetic beads, washed to remove nonspecific binding, and digested with proteinase K. Enriched m6A‐modified RNA was extracted for RT‐qPCR analysis.

### 2.21 Statistical Analysis

Data analysis was performed using GraphPad Prism 9 and SPSS Statistics 29. Results are expressed as mean ± standard deviation (SD). Normality was assessed using Shapiro–Wilk testing: normally distributed data were compared using Student's *t*‐test or one‐way analysis of variance (ANOVA) with Tukey's post hoc test for multiple comparisons; non‐normally distributed data were analyzed using Mann–Whitney *U* test or Kruskal–Wallis test with Dunn's multiple comparisons test.

## 3 Results

### 3.1 EA Ameliorates Ovarian Structure and Function in Mice With POI

**FIGURE 1:**
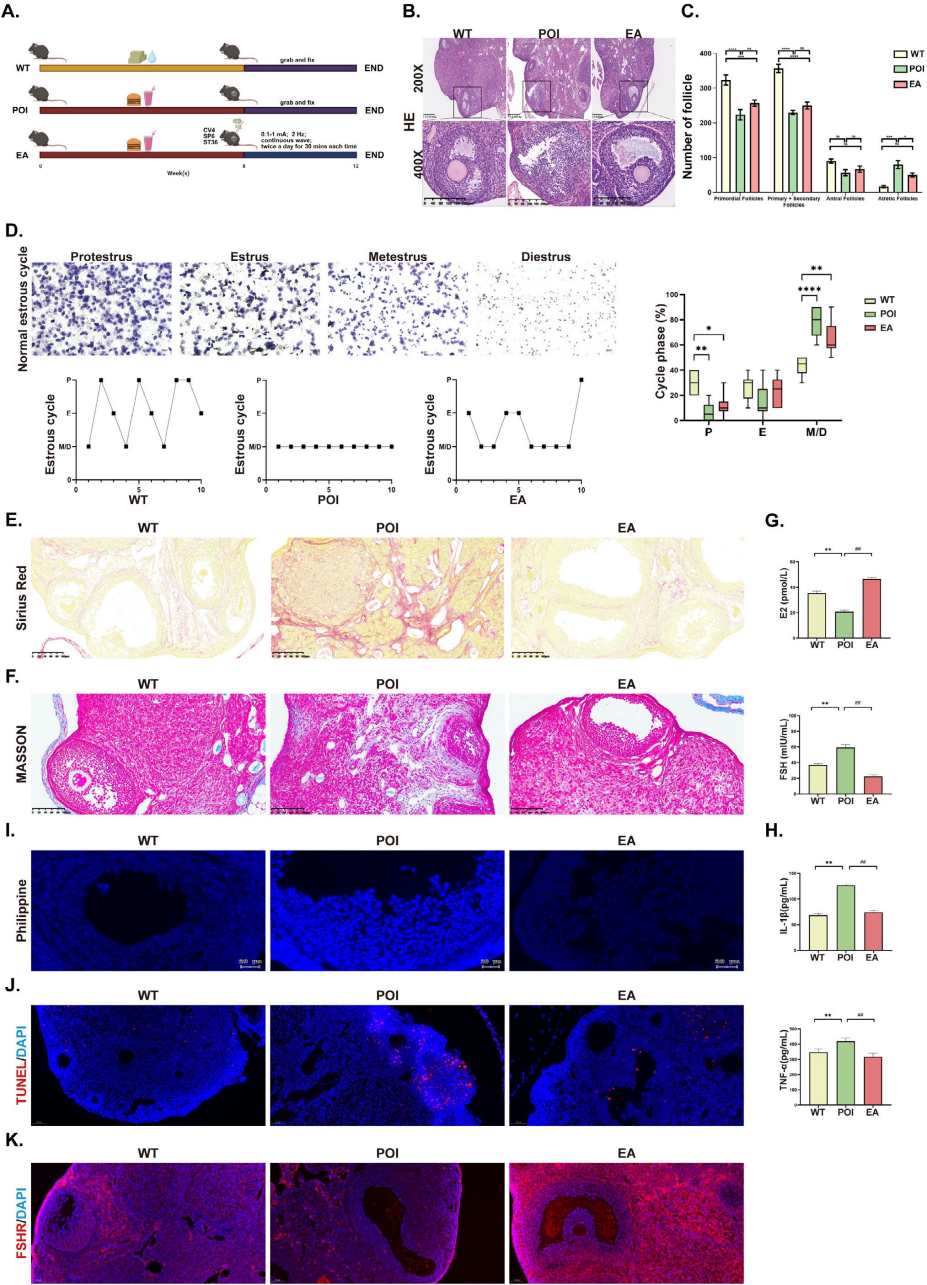
Electroacupuncture ameliorates ovarian structure and function in POI mice. (A) Timeline for POI model establishment and EA intervention. (B) HE staining of primordial, primary + secondary, antral, and atretic follicles (200×, 400×; *n* = 12). (C) Quantification of primordial, primary + secondary, antral, and atretic follicles (*n* = 12). (D) Estrous cycle phases assessed by crystal violet‐stained vaginal smears (100×, *n* = 6); POI vs. WT **p < 0.01; EA vs. WT *p < 0.05; POI vs. WT ****p < 0.0001; EA vs. WT **p < 0.01. (E) Sirius Red staining of ovarian fibrosis (200×; *n* = 12). (F) Masson's trichrome staining of ovarian fibrosis (200×; *n* = 12). (G) Serum E2 and FSH levels (*n* = 12); **p < 0.01 POI vs. WT; ^##^ *p* < 0.01 EA vs. POI. (H) Serum IL‐1β and TNF‐α levels (*n* = 12); **p < 0.01 POI vs. WT; ^##^ *p* < 0.01 EA vs. POI. (I) Filipin staining of granulosa cell total cholesterol content (200×; *n* = 12). (J) TUNEL staining of granulosa cell apoptosis (200×; *n* = 12). (K) Immunofluorescence of FSHR expression in granulosa cells (200×; *n* = 12). Panel A was created with BioRender.com.

The HFHS diet‐induced POI mouse model was established, and EA was administered at Guanyuan (CV4), bilateral Sanyinjiao (SP6), and Zusanli (ST36) acupoints to evaluate therapeutic efficacy (Figure 1A). HE staining revealed a significant decrease in primordial follicles and primary + secondary follicles, as well as an obvious increase in atretic follicles in POI group ovaries compared to WT, confirming successful modeling (Figure 1B,C).

Vaginal cytology demonstrated regular 3–4 day estrous cycles in WT mice, progressing through proestrus (predominantly nucleated epithelial cells with few leukocytes), estrus (cornified epithelial cells), metestrus (mixed epithelial cells and leukocytes), and diestrus (predominantly leukocytes). POI mice predominantly remained in metestrus or diestrus without completing cycles, indicating reproductive dysfunction (Figure 1D). Masson's trichrome and Sirius Red staining showed elevated collagen deposition in POI ovarian stroma, suggesting fibrosis (Figure 1E,F). EA intervention normalized estrous cyclicity and improved ovarian morphology.

Hormonal analysis indicated significantly decreased serum E2 and elevated FSH in POI mice; both parameters were restored to near‐normal levels after EA (Figure 1G). Additionally, EA effectively reduced the increased serum IL‐1β and TNF‐α levels observed in POI mice (Figure 1H), demonstrating anti‐inflammatory effects.

Filipin staining revealed substantial cholesterol accumulation in POI granulosa cells, which was significantly reduced by EA intervention (Figure 1I). TUNEL staining indicated elevated apoptosis in POI granulosa cells, attenuated after EA treatment (Figure 1J). Immunofluorescence showed downregulated follicle‐stimulating hormone receptor (FSHR) expression in POI ovaries, which EA rescued (Figure 1K), suggesting EA may inhibit granulosa cell apoptosis through FSHR upregulation.

Collectively, EA restored ovarian structure and function in HFHS‐induced POI mice by promoting folliculogenesis, normalizing estrous cycles, suppressing granulosa cell apoptosis, rebalancing sex hormones, and attenuating ovarian inflammation.

### 3.2 Single‐Cell Sequencing Reveals Aberrant Autophagy Activity in Granulosa Cells of POI Mice

**FIGURE 2:**
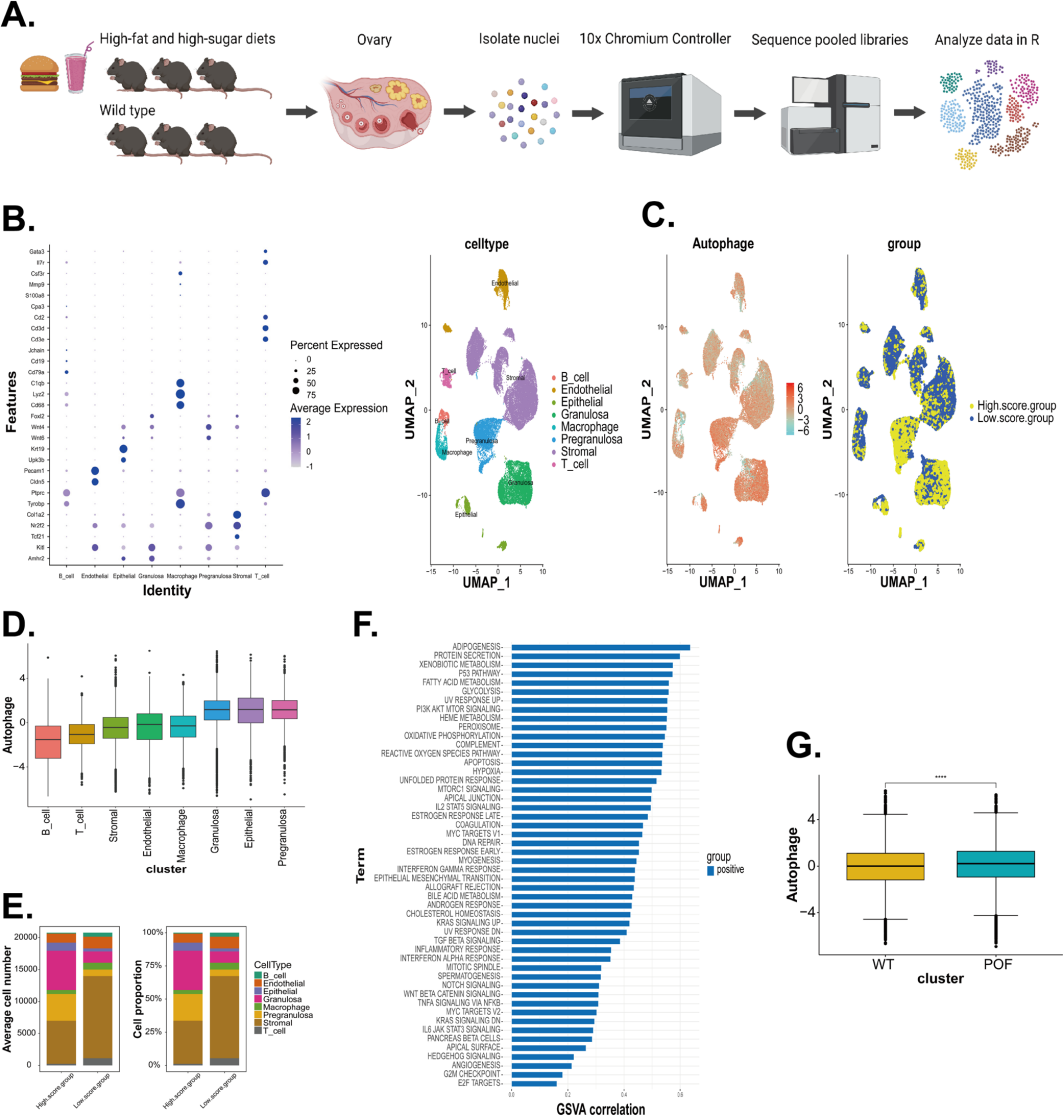
Granulosa cells are the primary ovarian cell subpopulation prone to autophagy. (A) Workflow for single‐cell analysis of ovarian tissues from HFHS diet‐induced POI mice. (B) Annotation of ovarian cell subpopulations in HFHS diet‐induced POI mice. (C) Autophagy‐related gene scoring across all cell subpopulations, with subpopulations classified into high‐ and low‐autophagy scoring groups. (D) Autophagy scores for each ovarian cell type. (E) Cell counts and proportions for each cell type within high‐ and low‐autophagy scoring groups. (F) Correlation analysis between granulosa cells and autophagy‐related pathways. (G) Autophagy scores of ovarian cells in POI versus WT groups. ****p < 0.0001 POI vs. WT. Panel A was created with BioRender.com.

ScRNA‐seq was performed on ovarian tissues from HFHS‐induced POI mice following the workflow illustrated in Figure 2A and Figures S1 and S2. After quality control and unsupervised clustering, ovarian cells were classified into distinct subpopulations based on established marker genes (Figure 2B). Autophagy‐related gene scores were subsequently calculated across all identified cell types, allowing stratification into high‐ and low‐autophagy activity groups (Figure 2C). Marked heterogeneity in autophagy scores was observed among ovarian cell populations, with granulosa cells exhibiting the most pronounced alterations in autophagy‐related transcriptional activity (Figure 2D). Consistently, both the number and relative proportions of ovarian cell subtypes differed significantly between the high‐ and low‐autophagy score groups (Figure 2E). GSVA correlation analysis further revealed significant positive associations between granulosa cells and multiple functional pathways, primarily involving energy and lipid metabolism, stress and inflammatory responses, apoptosis and survival regulation, hormone‐responsive signaling, as well as cell cycle and proliferation control. These findings indicate extensive functional reprogramming of granulosa cells during HFHS‐induced POI (Figure 2F). HALLMARK enrichment analysis demonstrated that inflammation‐ and stress‐related pathways, including P53_PATHWAY, TNFA_SIGNALING_VIA_NFKB, and IL6_JAK_STAT3_SIGNALING, were significantly upregulated in granulosa cells, whereas estrogen‐responsive pathways (ESTROGEN_RESPONSE_EARLY and ESTROGEN_RESPONSE_LATE) were markedly downregulated (Figure S3). KEGG pathway analysis revealed that ovarian cell subpopulations were enriched in signaling pathways such as PI3K–Akt and PPAR, both of which are closely involved in the regulation of granulosa cell autophagy. In parallel, the steroid hormone biosynthesis pathway was strongly associated with granulosa cell function, implicating its role in maintaining organelle homeostasis and hormone production during steroidogenesis. GO enrichment analysis further confirmed that granulosa cells are critically involved in female gonadal development and follicular maturation (Figure S4). Cell–cell interaction analysis indicated extensive communication among ovarian cell subpopulations, with granulosa cells exhibiting prominent interactions with multiple cell types that may cooperatively regulate the initiation and progression of autophagy (Figure S5). Pseudotime trajectory analysis further supported close functional and developmental associations among these cell populations (Figure S6). Moreover, comparison between the POI and WT groups revealed a significant difference in ovarian cell autophagy scores at the single‐cell level (Figure 2G, ****p < 0.0001), highlighting pronounced autophagy dysregulation in the POI model.

### 3.3 Electroacupuncture Suppresses Excessive Autophagy in Ovarian Granulosa Cells of POI Mice

**FIGURE 3:**
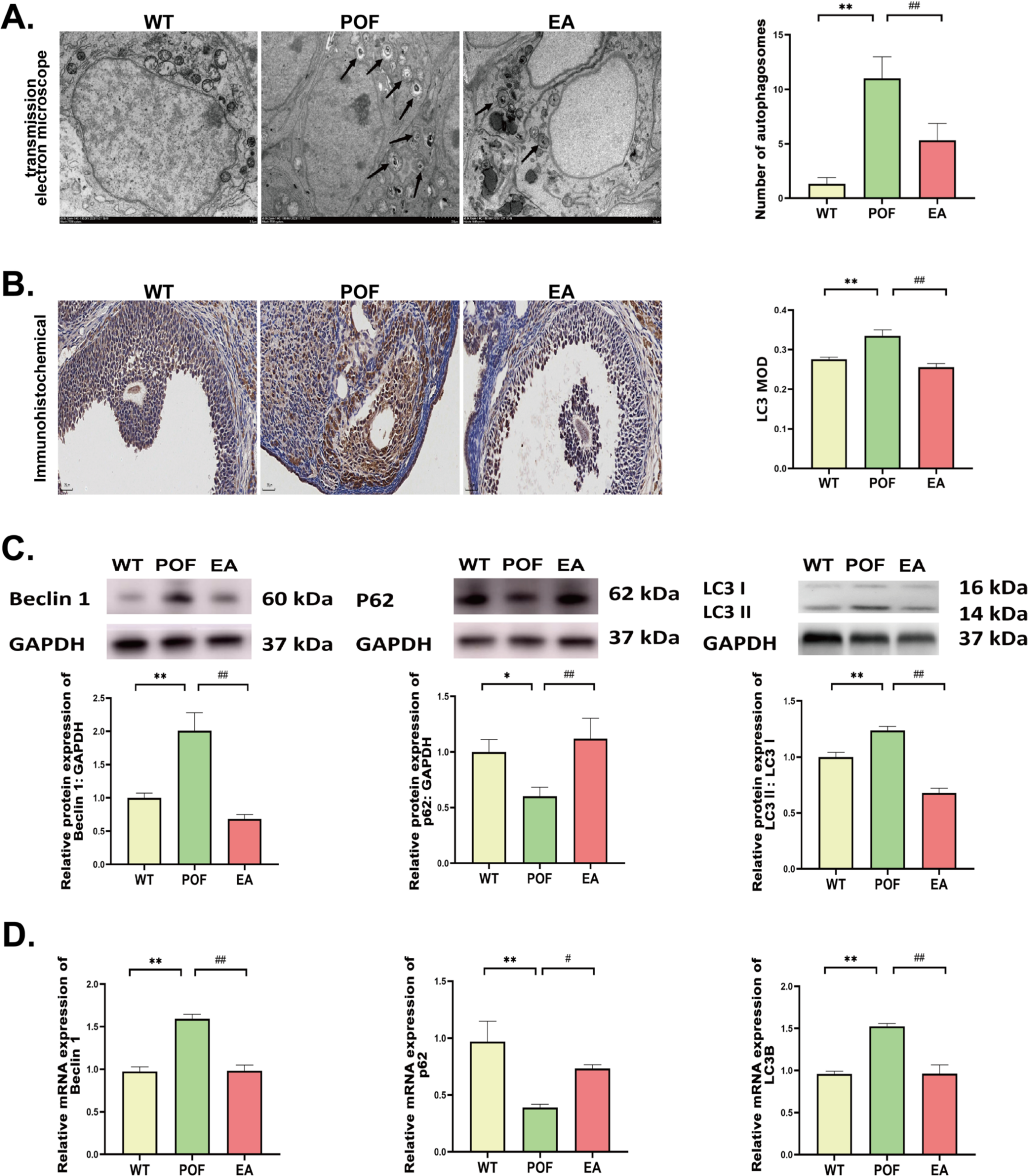
Electroacupuncture suppresses ovarian autophagy in POI mice. (A) TEM of autophagic structures (5000×; *n* = 3). Arrows indicate autophagosomes; **p < 0.01 POI vs. WT; ^##^ *p* < 0.01 EA vs. POI. (B) IHC of LC3 protein expression in granulosa cells (400×; *n* = 3); **p < 0.01 POI vs. WT; ^##^ *p* < 0.01 EA vs. POI. (C) Western blot of Beclin1, P62, and LC3 protein expression (*n* = 3); *p < 0.05, **p < 0.01 POI vs. WT; ^##^ *p* < 0.01 EA vs. POI. (D) RT‐qPCR of Beclin1, P62, and LC3B mRNA expression (*n* = 3); **p < 0.01 POI vs. WT; ^#^ *p* < 0.05, ^##^ *p* < 0.01 EA vs. POI.

TEM revealed ultrastructural alterations in ovarian granulosa cells across groups. POI group granulosa cells exhibited abundant autophagosome accumulation and disrupted mitochondrial integrity, indicating hyperactivated autophagy with organelle damage. In contrast, EA intervention markedly reduced autophagosome numbers and restored mitochondrial structural integrity, demonstrating mitigation of autophagy‐related organellar injury (Figure 3A). IHC staining confirmed expression changes of key autophagy regulators in granulosa cells. LC3 expression was significantly elevated in POI group granulosa cells but substantially reduced by EA treatment (Figure 3B). At molecular levels, Western blot analysis showed upregulated Beclin1 protein, downregulated P62, and increased LC3‐II/LC3‐I ratio in POI ovaries. RT‐qPCR corroborated these findings with elevated Beclin1 and LC3B mRNA alongside decreased P62 mRNA. EA intervention reversed these abnormalities: Beclin1 protein/mRNA decreased, P62 protein/mRNA increased, and LC3‐II/LC3‐I ratio declined (Figure 3C,D). Collectively, EA ameliorates ovarian microenvironment by suppressing excessive granulosa cell autophagy through coordinated downregulation of Beclin1/LC3 and upregulation of P62, thereby favoring follicular development.

### 3.4 Electroacupuncture Suppresses Granulosa Cell Autophagy via METTL3‐Mediated m6A Modification

**FIGURE 4:**
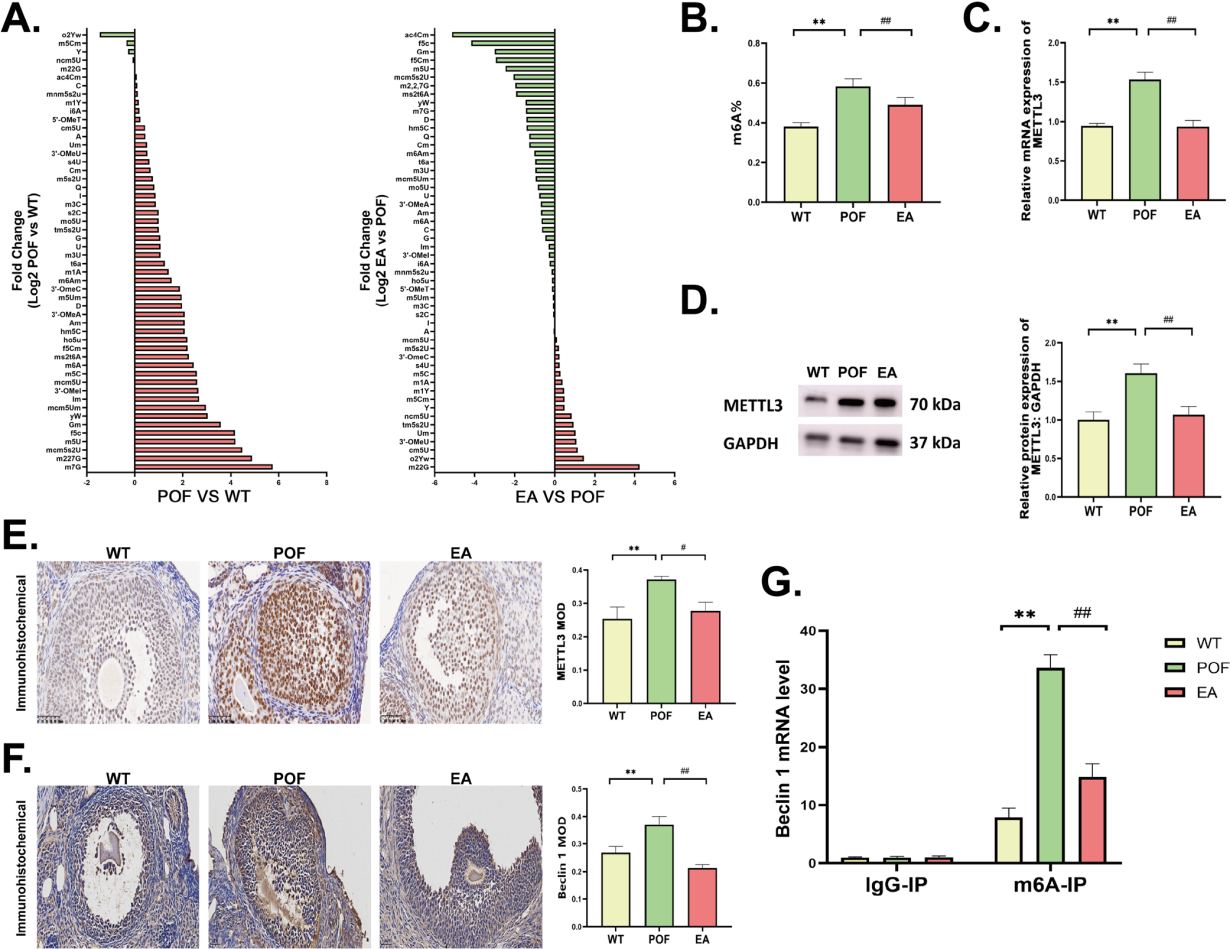
Electroacupuncture inhibits METTL3‐mediated m^6^A methylation of Beclin1 mRNA in POI mice. (A) LC–MS analysis of mRNA modifications in ovarian tissues across groups. (B) Colorimetric quantification of total m6A methylation levels (*n* = 4); **p < 0.01 POI vs. WT; ^##^ *p* < 0.01 EA vs. POI. (C) RT‐qPCR of METTL3 mRNA expression (*n* = 3); **p < 0.01 POI vs. WT; ^##^ *p* < 0.01 EA vs. POI. (D) Western blot of METTL3 protein expression (*n* = 3); **p < 0.01 POI vs. WT; ^##^ *p* < 0.01 EA vs. POI. (E) IHC of METTL3 protein in granulosa cells (400×; *n* = 3); **p < 0.01 POI vs. WT; ^#^ *p* < 0.05 EA vs. POI. (F) IHC of Beclin1 protein in granulosa cells (400×; *n* = 3); **p < 0.01 POI vs. WT; ^##^ *p* < 0.01 EA vs. POI. (G) MeRIP analysis of METTL3‐Beclin1 mRNA interaction (*n* = 3); **p < 0.01 POI vs. WT; ^##^ *p* < 0.01 EA vs. POI.

m6A—the most prevalent mammalian mRNA modification—dynamically regulates mRNA splicing, stability, and translational efficiency through reversible epigenetic mechanisms (Fu et al. 2014). LC–MS analysis of ovarian mRNA modifications revealed elevated levels of multiple mRNA modifications in POI mice, which EA intervention effectively normalized (Figure 4A). Colorimetric quantification further confirmed increased total m6A methylation in POI ovaries, significantly reduced by EA treatment (Figure 4B), indicating EA modulates ovarian m6A epitranscriptomic profiles.

Methyl transferase METTL3 has been extensively reported in gynecological disorders, such as influencing pregnancy outcomes by regulating placental function (Li et al. 2026) or participating in polycystic ovary syndrome (PCOS) development through modulating the Onecut2 factor (Sui et al. 2026). Based on this, the present study focused on the expression and role of METTL3 in POI. RT‐qPCR results revealed that METTL3 mRNA expression was upregulated in POI ovarian tissue, while EA intervention suppressed its expression (Figure 4C). Western blot and immunohistochemistry results further confirmed elevated METTL3 protein expression in POI, with EA treatment reversing this trend (Figure 4D,E). Collectively, these findings suggest pathological activation of METTL3 in POI, and EA may exert therapeutic effects by downregulating METTL3.

As Beclin1—a core autophagy initiator—upregulation correlates with autophagy activation (Liang et al. 1998), we investigated its role as an m6A target. Immunohistochemistry revealed increased Beclin1 protein in POI granulosa cells, suppressed by EA (Figure 4F). MeRIP‐PCR confirmed direct METTL3 binding to Beclin1 mRNA, with EA weakening this interaction. Reduced m6A methylation destabilized Beclin1 mRNA structure and diminished its translation (Figure 4G), identifying Beclin1 as a functional target of METTL3‐mediated m6A modification.

Collectively, these findings support a mechanism where METTL3‐mediated m6A hypermethylation stabilizes Beclin1 mRNA and enhances its translation, exacerbating granulosa cell autophagy in POI. EA counteracts this by downregulating METTL3 expression, attenuating m6A modification of Beclin1, and reducing Beclin1 protein levels, thereby suppressing pathological autophagy and restoring ovarian homeostasis.

## 4 Discussion

HFHS diets impose sustained metabolic and oxidative stress on ovarian tissues, leading to lipid peroxidation, mitochondrial dysfunction, inflammatory activation, and endocrine disruption, ultimately contributing to POI. Previous studies have demonstrated that HFHS exposure elevates ovarian oxidative stress while reducing antioxidant defenses such as SOD, catalase, and ATP, thereby impairing ovarian structure and function (Liu, Jing, et al. 2021; Zhu et al. 2021). Consistent with these findings, our HFHS‐induced POI model exhibited disrupted estrous cyclicity, follicular depletion, stromal fibrosis, inflammatory cytokine elevation, and abnormal gonadotropin–estrogen balance, collectively indicating severe ovarian dysfunction. Importantly, these pathological alterations were accompanied by excessive activation of autophagy in granulosa cells, suggesting that dysregulated autophagy represents a critical downstream effector of HFHS‐induced ovarian injury.

Substantial evidence supports a causal association between HFHS diets and POI development Hatch et al. 2018. Epidemiological data indicate that women consuming ≥ 7 sugary beverages per week exhibit significantly reduced pregnancy rates (Hatch et al. 2018). Experimental studies further show that HFHS diets impair meiotic spindle formation, disrupt mitochondrial function, elevate oxidative stress, and hinder follicular differentiation (Saben et al. 2016; Boots et al. 2016). In line with these reports, EA intervention in our study effectively reversed HFHS‐induced ovarian dysfunction. EA restored ovarian morphology, increased the proportion of mature follicles, reduced collagen deposition, normalized serum E_2_ and FSH levels, and suppressed ovarian IL‐1β and TNF‐α expression, indicating partial recovery of hypothalamic–pituitary–ovarian axis feedback and a rebalanced immune–endocrine microenvironment. At the cellular level, EA attenuated granulosa cell apoptosis—a known driver of follicular atresia (Choi et al. 2014; Choi et al. 2011)—while upregulating FSHR expression and reducing cholesterol accumulation, thereby enhancing follicular responsiveness and survival.

Granulosa cells are central regulators of folliculogenesis, steroidogenesis, and ovarian microenvironment homeostasis (Ford et al. 2020; Rimon‐Dahari et al. 2016). Under HFHS‐induced oxidative stress, granulosa cells undergo both apoptosis and excessive autophagy, which synergistically accelerate follicular atresia and ovarian aging (Shen et al. 2017). Our single‐cell RNA sequencing analysis identified granulosa cells as the most autophagy‐active ovarian subpopulation in POI mice. Moreover, autophagy scores in granulosa cells strongly correlated with senescence‐related signaling pathways, highlighting autophagy dysregulation as a key determinant of granulosa cell fate under metabolic stress. These findings position granulosa cells as the primary cellular target of HFHS‐induced injury and EA‐mediated protection.

Autophagy is a tightly regulated multistep process involving ULK1/2–ATG13–FIP200 initiation complexes, VPS34–Beclin1‐mediated phagophore nucleation, and ATG5–ATG12–ATG16L/LC3‐II–dependent autophagosome maturation (Prerna and Dubey 2022). Among these components, Beclin1 functions as a central molecular hub integrating upstream metabolic and stress signals and initiating autophagosome formation as a core component of the VPS34 complex (Li et al. 2022). The PI3K/AKT/mTOR pathway negatively regulates autophagy by suppressing Beclin1 activity, and VEGFA has been reported to protect ovarian function by activating PI3K/AKT/mTOR signaling and restraining excessive autophagy (Dai et al. 2023). Under sustained HFHS‐induced metabolic stress, this inhibitory control appears to be disrupted, favoring persistent Beclin1‐driven autophagy activation.

A major novel contribution of this study is the identification of RNA epigenetic regulation as an upstream mechanism governing Beclin1‐mediated autophagy in granulosa cells. Increasing evidence implicates m6A RNA methylation in ovarian dysfunction and POI pathogenesis (Zhang et al. 2025). METTL3, the core m6A methyltransferase, not only catalyzes m6A deposition but also enhances mRNA stability and translational efficiency (Zhang, Yu, et al. 2021). In the present study, HFHS feeding significantly increased global m6A methylation levels in ovarian tissues, accompanied by upregulation of METTL3 expression. Importantly, RIP‐qPCR and LC–MS analyses revealed increased m6A enrichment on Beclin1 mRNA, suggesting that METTL3 preferentially targets autophagy‐related transcripts under POI conditions. This enhanced m6A modification was associated with increased Beclin1 mRNA stability and protein expression, providing a mechanistic explanation for the sustained autophagy activation observed in granulosa cells at both single‐cell and bulk tissue levels.

EA intervention effectively counteracted these epigenetic and autophagic alterations. EA reduced METTL3 expression, decreased m6A modification of Beclin1 mRNA, downregulated Beclin1 and LC3‐II expression, and restored P62 accumulation, indicating attenuation of excessive autophagic flux. Transmission electron microscopy further confirmed a marked reduction in autophagosome abundance in granulosa cells following EA treatment. Collectively, these findings support a model in which EA alleviates HFHS‐induced POI by suppressing METTL3‐dependent m6A methylation of Beclin1 mRNA, thereby restraining pathological autophagy activation.

Although direct genetic or pharmacological manipulation of METTL3 was not performed, the coordinated changes in METTL3 expression, Beclin1 m6A methylation, autophagy‐related factors, and ovarian functional outcomes provide strong functional association evidence. These findings are consistent with previous reports demonstrating that METTL3‐mediated m6A methylation promotes the translation of autophagy‐related genes under stress‐related pathological conditions (Zhang et al. 2025). Accordingly, we propose a METTL3–m6A–Beclin1–autophagy regulatory axis as a key epigenetic mechanism linking HFHS‐induced metabolic stress to granulosa cell dysfunction and follicular atresia in POI.

It should be emphasized that this axis represents a putative mechanistic framework rather than a fully validated signaling cascade. The absence of METTL3 inhibition, Beclin1 knockdown, or pharmacological autophagy modulation limits causal inference. Nevertheless, accumulating evidence indicates that EA can influence RNA epigenetic modifications, including m6A and ac4C, through mechanisms such as regulation of methyltransferase activity, NAT10 inhibition, or alterations in cellular SAM metabolism (Geng, Liu, et al. 2023). In this context, our findings suggest that EA may protect ovarian function by modulating epigenetic–autophagy coupling in granulosa cells.

In summary, this study integrates HFHS‐induced metabolic stress, RNA epigenetic regulation, and autophagy into a unified mechanistic framework, highlighting METTL3‐mediated m6A modification of Beclin1 as a critical regulatory node in POI. Given the dynamic and reversible nature of RNA modifications, targeting this axis may offer novel therapeutic opportunities for POI intervention. Future studies should focus on validating causal relationships within this pathway and identifying upstream regulators of EA‐mediated METTL3 suppression, thereby advancing epigenetics‐guided acupuncture‐based strategies for ovarian aging and reproductive disorders.

## 5 Conclusion

**FIGURE 5:**
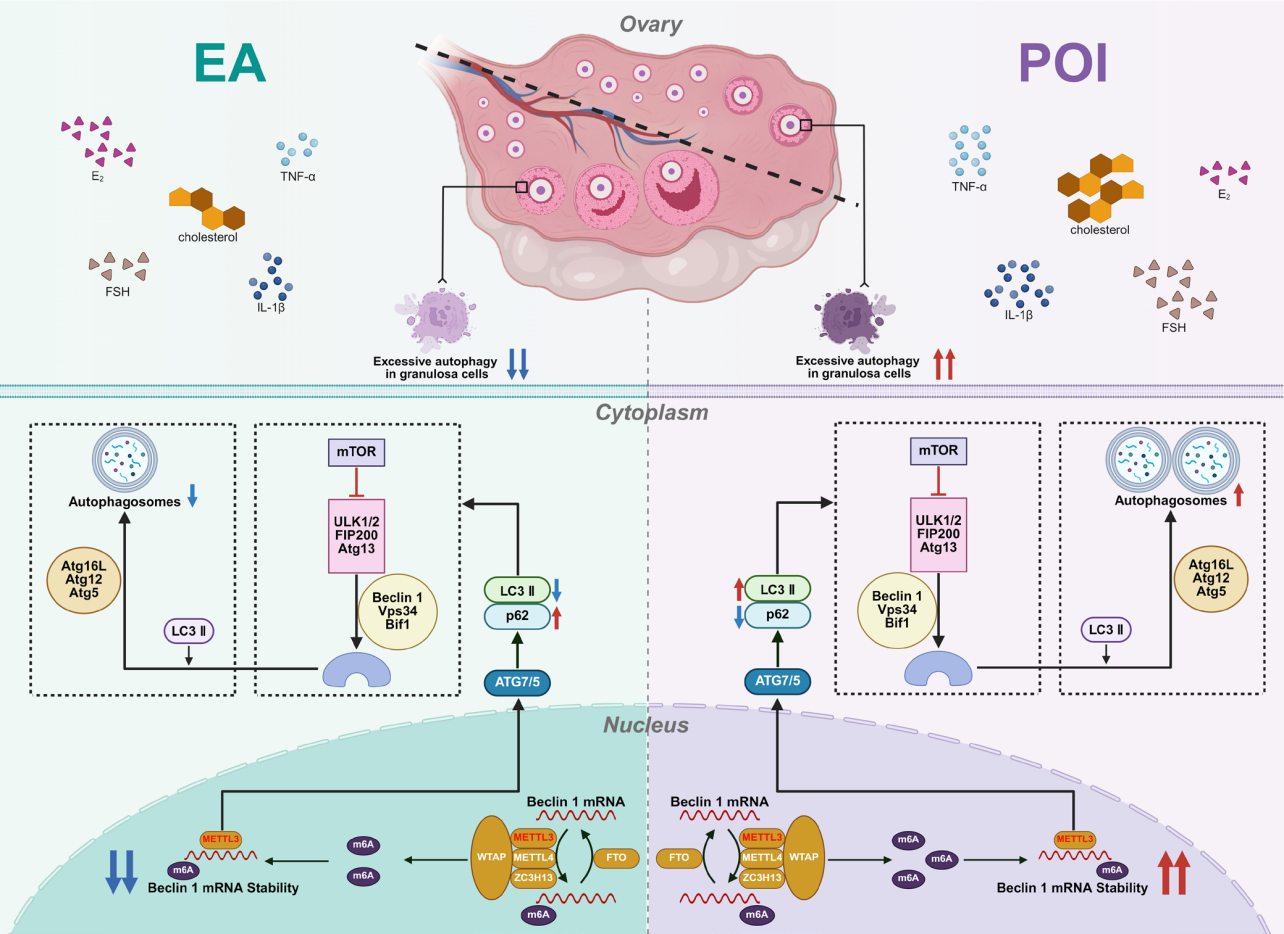
Proposed mechanism of HFHS‐induced POI and EA‐mediated rescue. HFHS diet activates excessive autophagy in ovarian granulosa cells by upregulating the methyltransferase METTL3, which enhances m6A modification levels on Beclin1 mRNA, consequently contributing to POI. EA intervention reverses these alterations, selectively suppressing pathological autophagy and restoring ovarian function. The figure was created with BioRender.com.

In conclusion, this study elucidates key molecular mechanisms by which a HFHS diet induces POI, establishing a pathological link between granulosa cell autophagy activation and m6A methylation. Our core finding demonstrates that EA therapy ameliorates ovarian dysfunction by downregulating the expression of methyltransferase METTL3. This specifically reduces m6A modification levels on the mRNA of the autophagy‐related gene Beclin1, consequently diminishing its transcript stability and translational efficiency, thereby suppressing excessive granulosa cell autophagy (Figure 5). Elucidating this mechanism not only advances understanding of epigenetic regulation in POI pathogenesis but also identifies METTL3 reduction as a potential therapeutic target, providing a mechanistic foundation for EA treatment of POI.

## Author Contributions

The study conception and design were contributed to by all authors. Lele Ling, Sining He, Xue Zhao, and Yaran Sheng contributed equally to this work. Lele Ling and Sining He conceptualized the study, designed and performed the experiments, analyzed the data, and wrote the original draft. Xue Zhao and Yaran Sheng conducted statistical analysis and edited the manuscript. Mengying Hong, Long Yuan, Peng Liu, and Boliang Ke performed data curation and visualization. Bingrong Li and Bimeng Zhang supervised the project and finalized the manuscript. All authors reviewed and approved the final version.

## Funding

This work was supported by the Science and Technology Key Research Program of Songjiang District, Shanghai (Medical and Health Sciences), Grant No. 2025SJKJGG004.

## Ethics Statement

Ethical approval of this animal experiment was granted by the Experimental Animal Center of Shanghai General Hospital affiliated to Shanghai Jiao Tong University School of Medicine.

## Consent

The authors have nothing to report.

## Conflicts of Interest

The authors declare no conflicts of interest.

## Supporting information


**Figure S1:** Top 10 differentially expressed marker genes in each cell subpopulation identified by scRNA‐seq.
**Figure S2:** Quality control, filtering, and batch effect correction of scRNA‐seq data.
**Figure S3:** Correlation analysis of HALLMARK pathways and GSVA scores across cell subpopulations identified by scRNA‐seq.
**Figure S4:** Functional enrichment analysis of cell subpopulations identified by scRNA‐seq.
**Figure S5:** Intercellular interaction analysis among cell subpopulations identified by scRNA‐seq.
**Figure S6:** Pseudotime trajectory analysis based on scRNA‐seq.

## Data Availability

The data that support the findings of this study are available from the corresponding author upon reasonable request.
