# Supplementary material for: Electroacupuncture Ameliorates Premature Ovarian Insufficiency by Inhibiting METTL3‐Mediated m6A Methylation of Beclin 1 mRNA in Granulosa Cells
Source: Food Sci Nutr. 2026 Jun 21;14(6):e71697. doi: 10.1002/fsn3.71697 (PMC13284094; doi:10.1002/fsn3.71697)
Supplement: Supplementary file 1 — Figure S1: Top 10 differentially expressed marker genes in each cell subpopulation identified by scRNA‐seq. Figure S2: Quality control, filtering, and batch effect correction of scRNA‐seq data. Figure S3: Correlation analysis of HALLMARK pathways and GSVA scores across cell subpopulations identified by scRNA‐seq. Figure S4: Functional enrichment analysis of cell subpopulations identified by scRNA‐seq. Figure S5: Intercellular interaction analysis among cell subpopulations identified by scRNA‐seq. Figure S6: Pseudotime trajectory analysis based on scRNA‐seq. [file FSN3-14-e71697-s001.docx]

Supplemental Figure
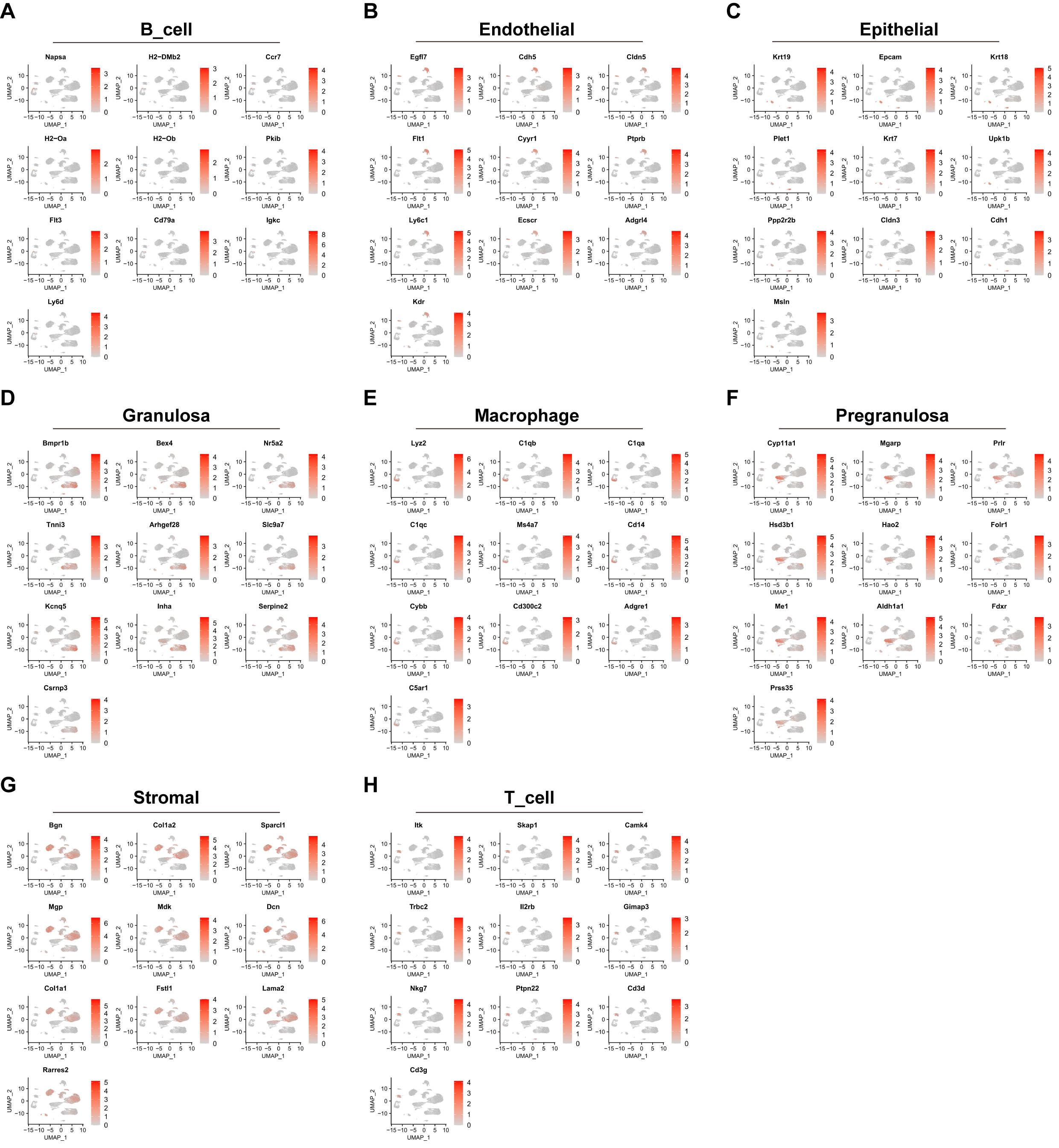


Fig.S1 Top 10 Differentially Expressed Marker Genes in Each Cell Subpopulation Identified by scRNA-seq


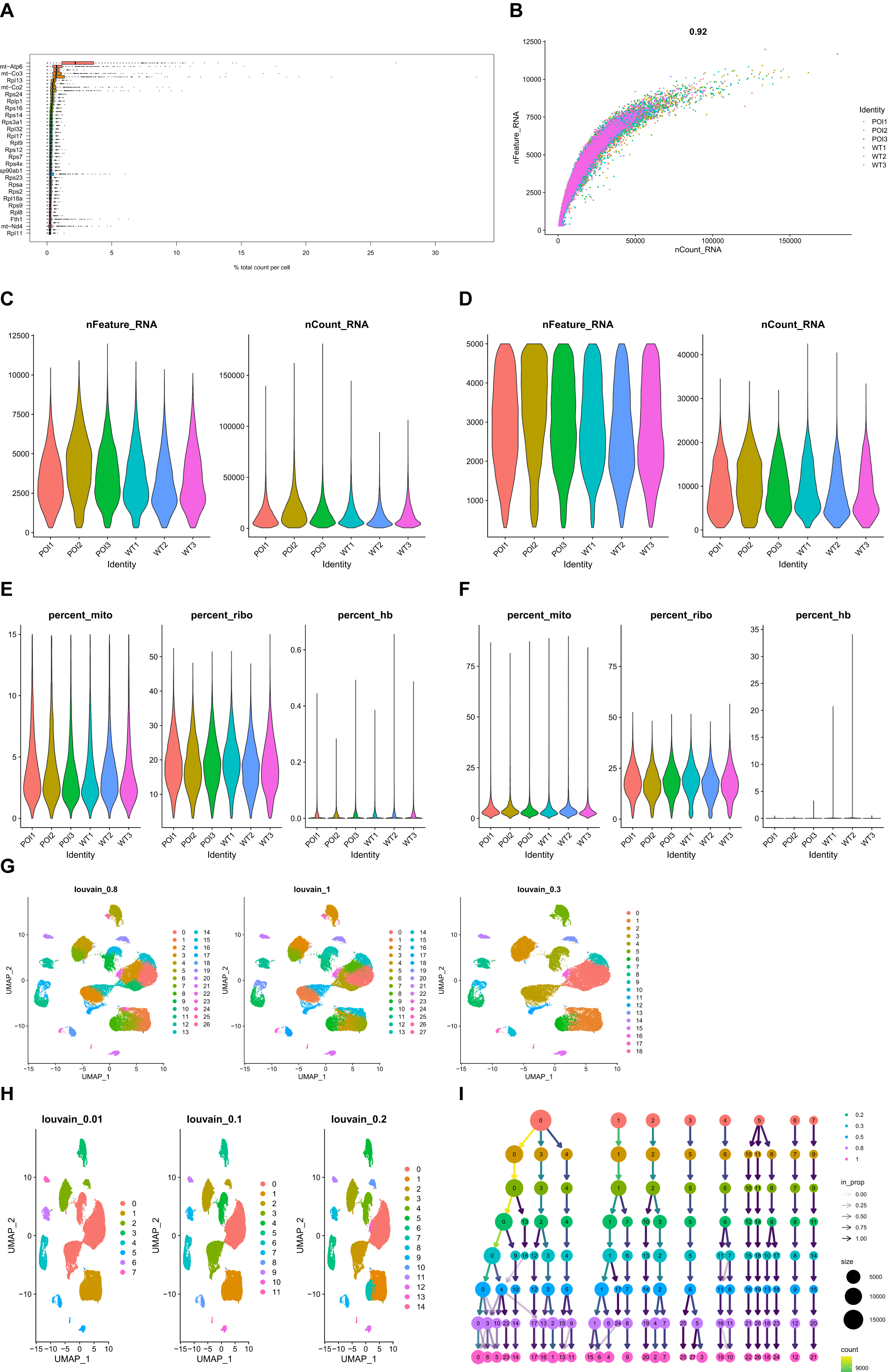


Fig.S2 Quality control, filtering, and batch effect correction of scRNA-seq data

A–F: Quality control filtering and downstream analysis of scRNA-seq data.

G–I: Removal of inter-sample batch effects in single-cell samples.


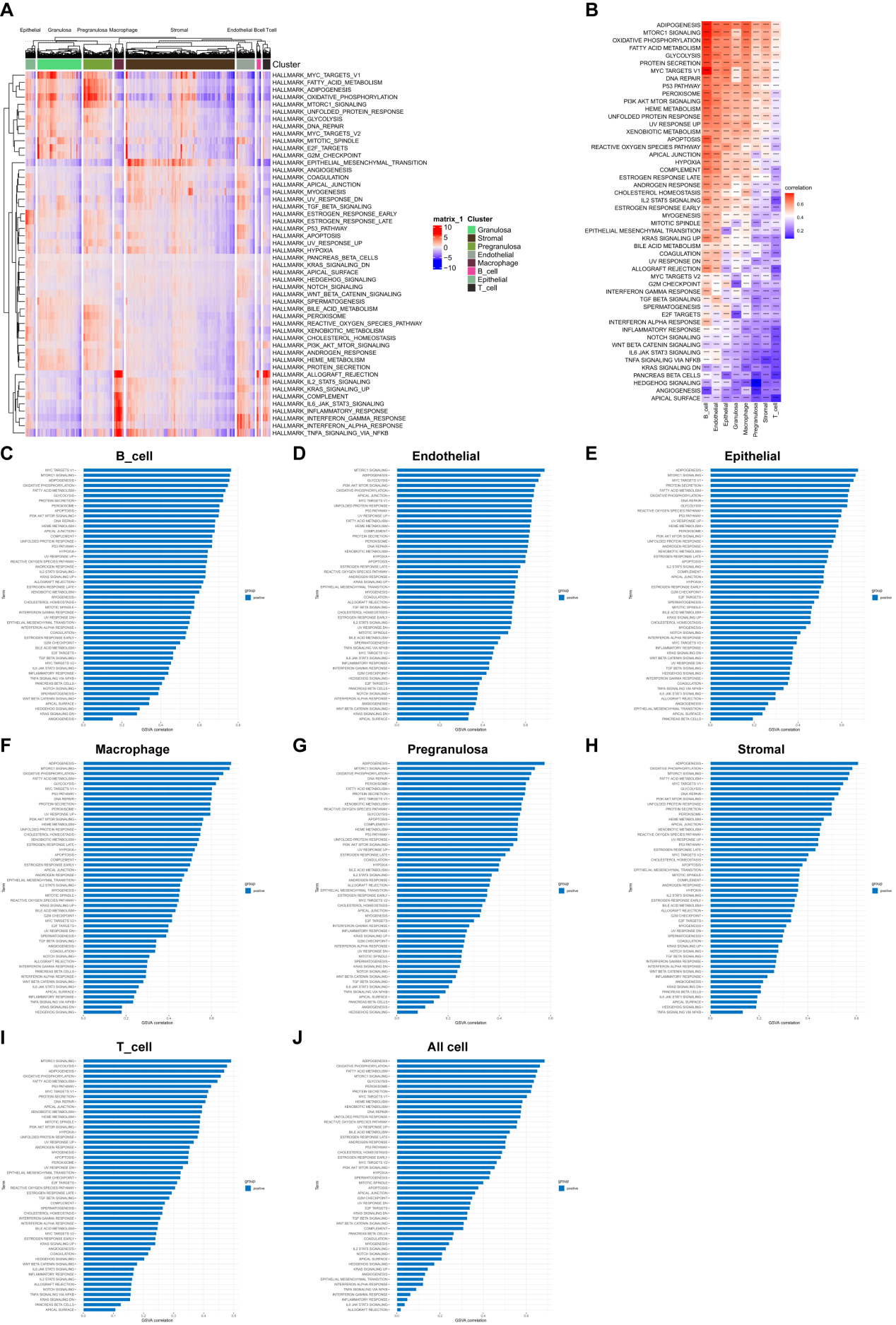


Fig.S3 Correlation Analysis of HALLMARK Pathways and GSVA Scores Across Cell Subpopulations Identified by scRNA-seq


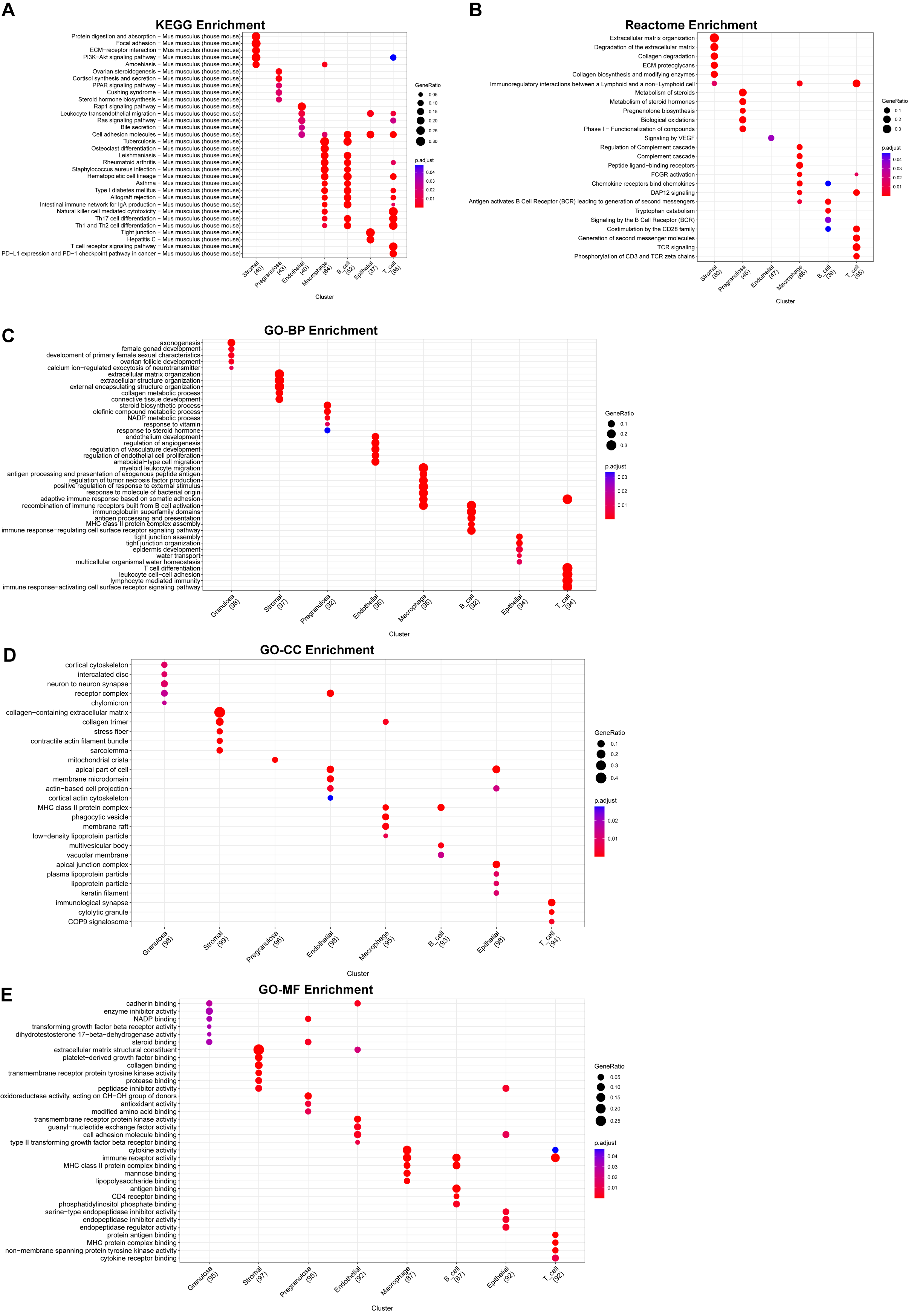


Fig.S4 Functional Enrichment Analysis of Cell Subpopulations Identified by scRNA-seq


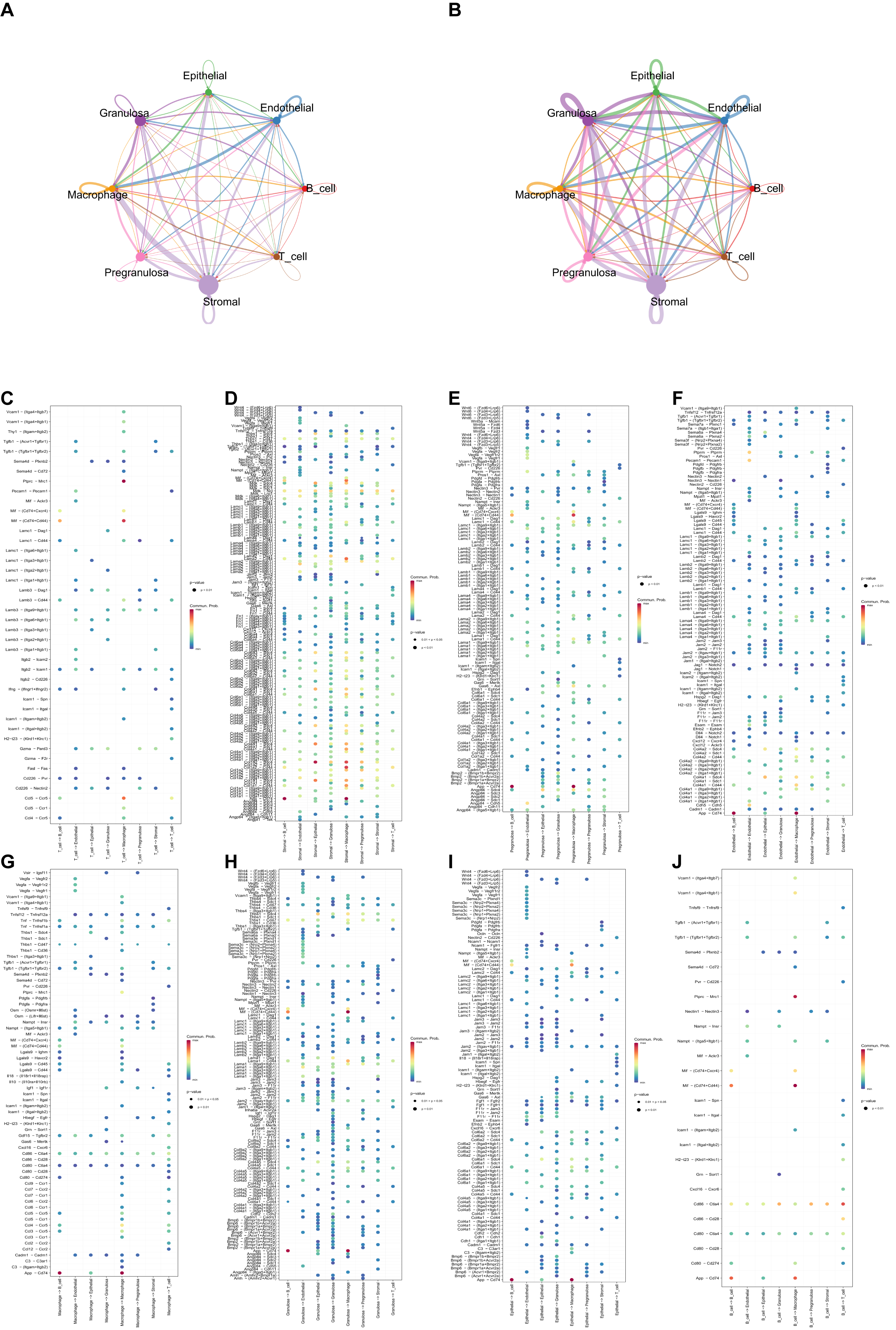


Fig.S5 Intercellular Interaction Analysis Among Cell Subpopulations Identified by scRNA-seq


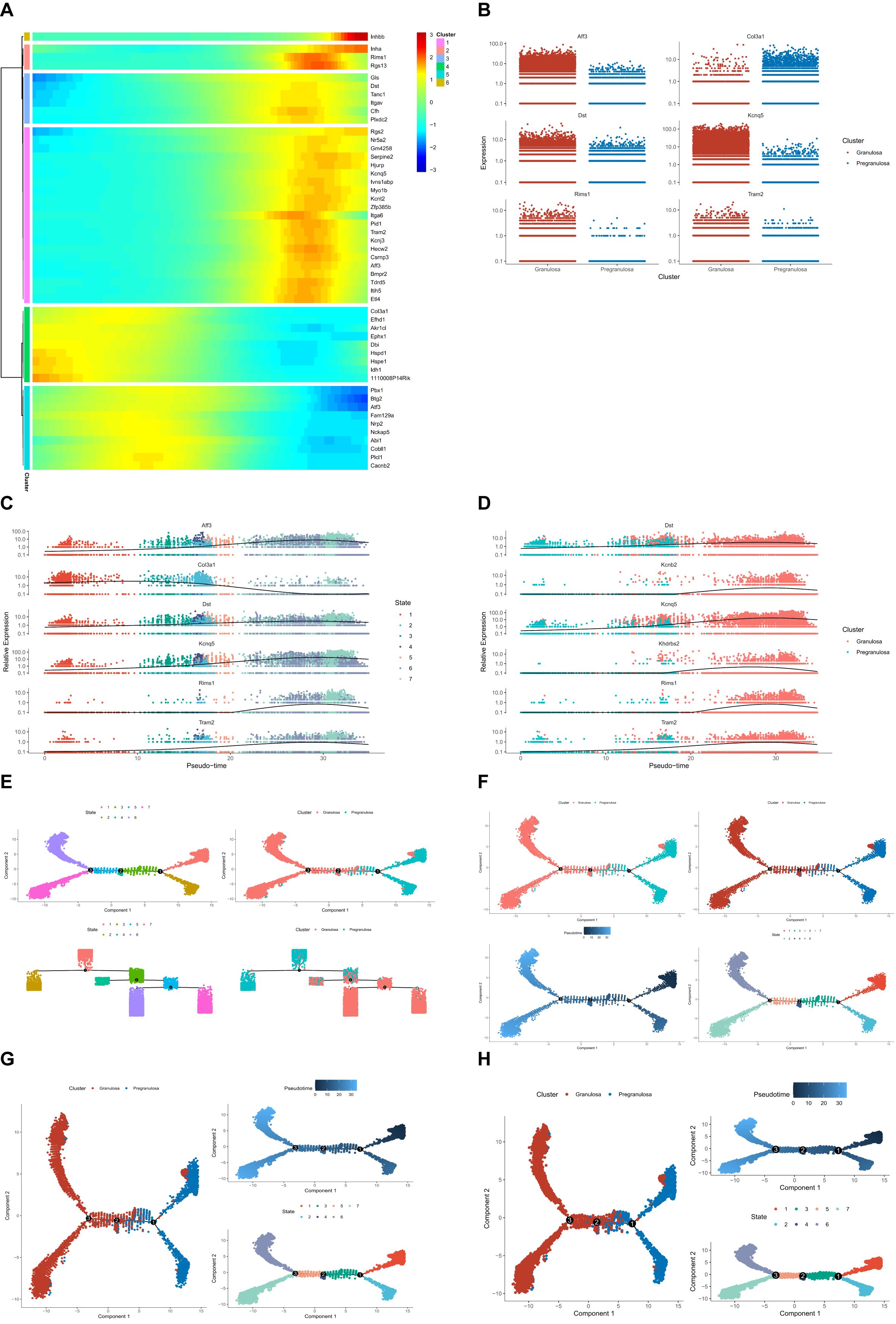


Fig.S6 Pseudotime Trajectory Analysis Based on scRNA-seq
